# Multi-interactive feature embedding learning for medical image segmentation

**DOI:** 10.3389/fmed.2025.1661984

**Published:** 2025-09-24

**Authors:** Yijia Huang, Yue Luo

**Affiliations:** ^1^School of Public Health, Chengdu University of Traditional Chinese Medicine, Chengdu, Sichuan, China; ^2^School of Intelligent Medicine, Chengdu University of Traditional Chinese Medicine, Chengdu, Sichuan, China

**Keywords:** medical image segmentation, self-supervised learning, adaptive feature modulation module, bi-directional fusion module, multi-branch vision mamba

## Abstract

Medical image segmentation task can provide the lesion object semantic information, but ignores edge texture details from the lesion region. Conversely, the medical image reconstruction task furnishes the object detailed information to facilitate the semantic segmentation through self-supervised learning. The two tasks are supplementary to each other. Therefore, we propose a multi-interactive feature embedding learning for medical image segmentation. In the medical image reconstruction task, we aim to generate the detailed feature representations containing rich textures, edges, and structures, thus bridging the low-level details lost from segmentation features. In particular, we propose an adaptive feature modulation module to efficiently aggregate foreground and background features to obtain a comprehensive feature representation. In the medical segmentation task, we propose a bi-directional fusion module fusing all important complementary information between the two tasks. Besides, we introduce a multi-branch visual mamba to capture structural information at different scales, thus enhancing model adaptation to different lesion regions. Extensive experiments on four datasets demonstrate the effectiveness of our framework.

## 1 Introduction

Medical image segmentation tasks (1–5) focus on extracting lesion regions from complex medical images, thereby assisting doctors to perform subsequent disease diagnosis, treatment planning and efficacy assessment. In particular, skin lesion segmentation and cell boundary detection tasks enable precise localization of key tissues or lesions, which supports in early diagnosis and clinical assisted decision making by visualizing lesion results (6). Therefore, in public health management, deep learning-based medical image segmentation methods can effectively improve the efficiency of group patient lesion detection. These methods can help public health departments to better monitor and predict the disease spread, thereby promoting disease prevention and treatment.

Existing medical segmentation methods (7, 8) construct complex network structures to improve performance, but ignore texture and boundary detail information about lesion regions in medical images. U-Net (9) introduces encoder-decoder structure, and designs skip connections to combine the different-level semantic information. UNet++ (10) adds dense jump paths and nested decoders to enhance multiscale feature learning. MFSNet (11) combines multi-scale feature extraction and attention mechanisms, which further improves segmentation performance. However, medical image segmentation task emphasizes on extracting high-level semantic features, resulting in the loss of pixel-level detail information. In contrast, the medical image reconstruction task can provide pixel-level detail information (e.g., texture and boundaries) to the medical image segmentation task through a self-supervised learning strategy, thus obtaining more accurate segmentation results.

Moreover, since convolutional neural network (CNN)—based segmentation methods (12–14) rely on local receptive fields and convolutional structures, it is difficult to effectively capture the non-local relations and structural ambiguity features present in the lesion region. Therefore, Transformer-based segmentation methods (15–18) aim to improve modeling ability for global context, thus enhancing semantic consistency and regional integrity. For example, TransUNet (19) combines the local feature learning of CNN and the global context learning of Transformer advantages. TransFuse (20) designs a two-branch network to capture local and global features, and then fuses them using a fusion module in the decoding stage. This architectural design enhances the model's capability to capture fine-grained boundaries and structural information, thereby improving segmentation accuracy. Although Transformer-based methods can help to recognize organ contours, lesion shapes, and spatial layouts by capturing distant dependencies in medical images through a self-attention mechanism, they require high computational and memory costs. Compared with Transformer-based architectures, Mamba (21, 22) offers lower computational overhead while maintaining strong long-sequence modeling and structural awareness. This is especially valuable in medical image segmentation, where accurate delineation of anatomical structures requires modeling long-range dependencies and preserving fine-grained spatial details. By efficiently extracting spatially hierarchical features, Mamba enables real-time and resource-constrained applications while ensuring precise boundary segmentation.

In this paper, we propose a multi-interactive feature embedding learning (MFEL) for medical image segmentation. Specifically, MFEL consists of a feature interaction-driven image reconstruction (FIIR) and a feature-embedded representation image segmentation (FRIS). On the one hand, FIIR reconstructs the foreground image, background image and medical image through self-supervised learning, thus extracting complete pixel-level features. In particular, an adaptive feature modulation module effectively enhances foreground and background feature representation via the learned modulation parameters, thereby obtaining a more comprehensive and fine-grained pixel-level feature information. On the other hand, FRIS aims to fuse the two different-level features between the reconstruction and segmentation tasks, thereby improving the performance of segmentation task. In particular, a bi-directional fusion module is designed to fuse the feature representations from two tasks, which enhances the information interaction. Moreover, a multi-branch vision mamba utilizes the parallel branching structure and linear state space modeling capability, improving model semantic understanding about different lesion regions.

Our contributions can be summarized as follows:

- We explore an MFEL framework between medical image reconstruction task and medical image segmentation task, and then achieve superior segmentation performance.
- An adaptive feature modulation module is proposed to construct modulation parameters from foreground and background features, thus obtaining a comprehensive pixel-level feature representation.
- A bi-directional fusion module is introduced to establish complementary relationships between structural details and deep semantics, thus enabling feature information interaction between two different-level tasks.
- Multi-branch vision mamba is designed to combine state-space modeling and multi-branch parallel mechanism, efficiently modeling the multi-scale structural information from lesion regions.

## 2 Related work

### 2.1 Medical image segmentation methods

Convolutional neural networks (CNNs) have achieved remarkable success in medical image segmentation by leveraging hierarchical representations and strong inductive biases (23–26). Recent methods enhance segmentation performance by integrating boundary cues and multi-scale features. DCSAU-Net (27) introduces a split attention mechanism with semantic retention, while U-Net v2 (28) incorporates boundary information to refine local detail representations. Transformer-based architectures address CNNs' limitations in modeling long-range dependencies. These models exhibit strong global context awareness and have demonstrated competitive performance in medical image segmentation (19, 29–35). CASF-Net (36) employs dual-branch modeling to combine global semantics and fine-grained features. CSWin-UNet (18) utilizes cross-shaped window attention to improve spatial interactions with low computational cost. Hybrid designs, such as TBConvL-Net (37) and MobileUNETR (38), further balance local detail extraction and global reasoning. Since medical image segmentation as a high-level vision task focuses on extracting semantic structural information, the pixel-level details are ignored. In contrast, we introduce the medical image reconstruction task to learn fine-grained feature representations through self-supervised learning, thus bridging the shortcomings from the semantic segmentation task.

### 2.2 Self-supervised learning methods

Self-supervised learning methods have been widely applied in tasks such as image reconstruction (39, 40), inpainting (41–43), and enhancement (44, 45). For example, Self-path (46) introduces a region-aware contrastive learning framework, which enforces consistency between local and global representations. This strategy effectively enhances feature discrimination and contextual modeling for downstream segmentation tasks. DSFormer (47), designed for multi-contrast MRI reconstruction, proposes a dual-domain self-supervised Transformer architecture. It performs joint reconstruction and context restoration in both k-space and image space, achieving collaborative modeling of structural information and significantly improving reconstruction quality and generalization. MiM (48) targets 3D medical image analysis by proposing a hierarchical Mask-in-Mask masking mechanism. Through a coarse-to-fine masking strategy combined with residual reconstruction, it guides the model to learn rich semantic structures and fine spatial details, thereby improving its adaptability to downstream tasks such as segmentation and classification. In contrast, the medical image reconstruction task guides the model to focus on pixel-level content (e.g., texture, structure, edges), thus compensating the loss of important details in the cell and skin lesion segmentation tasks.

### 2.3 Vision mamba

Mamba (21) is a novel sequence modeling architecture built upon State Space Models. It enables efficient inference while modeling long-range dependencies. Unlike traditional self-attention mechanisms, Mamba introduces learnable state space kernels and applies linear operations in a sliding-window manner. This design supports global modeling while significantly reducing computational complexity, achieving linear time and space costs. VMamba (22) extends Mamba to vision tasks by introducing a 2D Selective Scan mechanism, which aggregates spatial context from multiple directions with linear complexity, achieving superior accuracy and efficiency over Vision Transformers. Compared with CNNs, Mamba is not limited by local receptive fields and can capture global sequential and contextual information. Compared with Transformers, Mamba avoids the high computational overhead of self-attention in long sequences, achieving better efficiency and performance. These advantages make Mamba particularly suitable for high-resolution or 3D medical image tasks. In medical image segmentation (49–51), accurately capturing the spatial structure and contextual relationships of lesions is critical for performance. Mamba's strength in long-range modeling and computational efficiency provides strong support for this task. Recently, Mamba has been increasingly applied in medical scenarios. U-VM-UNet (52) integrates sparse gating and low-rank decomposition to design an efficient visual selective scan module, achieving strong segmentation results across datasets. Mamba-Sea (53) proposes a global-to-local sequence augmentation mechanism and builds a pure SSM-based framework, improving generalization in cross-domain segmentation tasks. VM-UNetV2 (54) combines Vision Mamba with the UNet v2 (28) architecture and introduces a semantic and detail injection module, showing better performance than conventional models in skin and polyp segmentation. SMM-UNet (55) constructs selective and multi-scale fusion Mamba modules to enhance feature representation at different scales while keeping the network compact. CAMS (56) completely removes convolution and attention mechanisms, adopting a pure Mamba encoder and dual decoder structure to balance global modeling and fine-grained detail recovery in cardiac image segmentation. Therefore, we adopt a multi-branch mamba structure to establish long-distance dependency capturing global relationships and effectively aggregating contextual information, thus enhancing global semantic representation.

## 3 Methods

**Figure 1:**
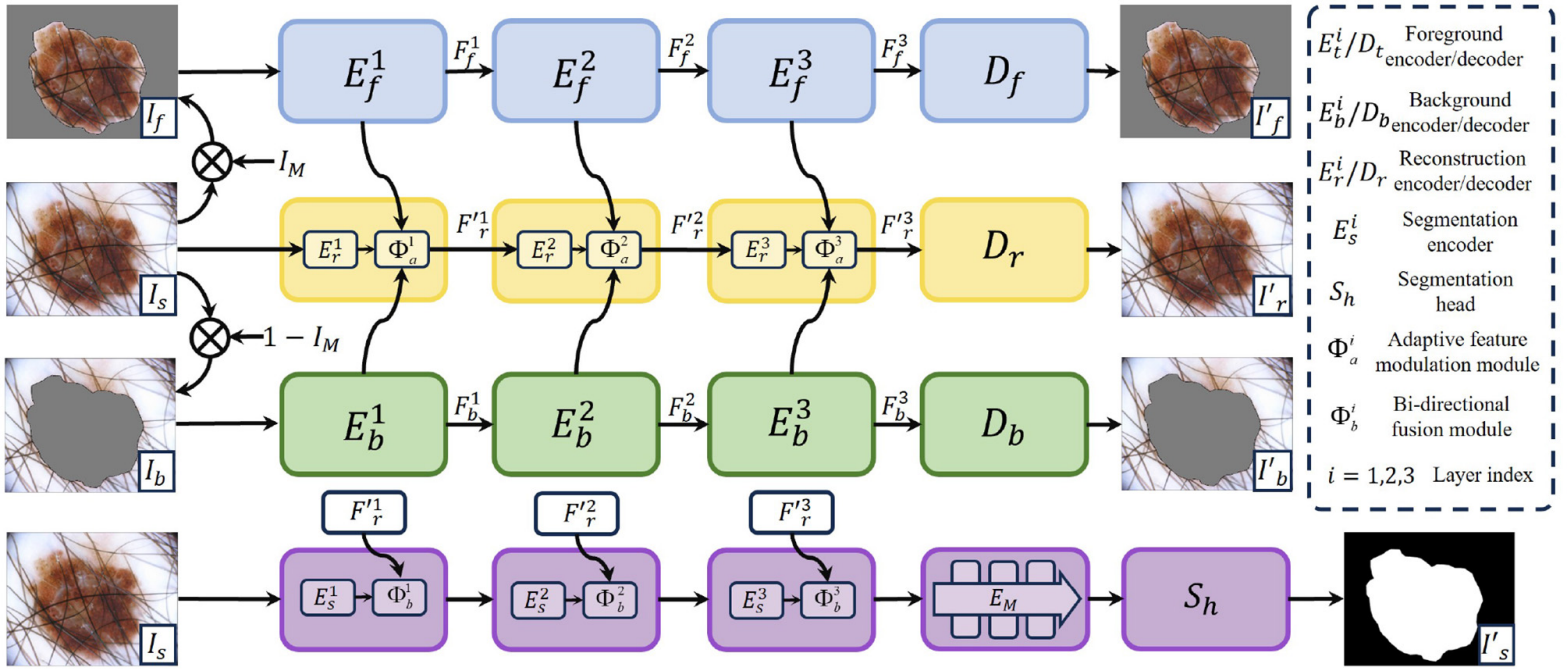
The overall framework illustration of the proposed MFEL. MFEL consists of FIIR and FRIS. FIIR aims to extract pixel-level features through self-supervised learning, thus helping the segmentation task to obtain finer-grained information. FRIS fuses semantic segmentation features and fine-grained reconstruction features to generate a more comprehensive feature representation.

Medical image segmentation task aims to extract the lesion object semantic information, but ignores the pixel-level detail information. In contrast, medical image reconstruction task focuses on mining low-level content information. Therefore, we combine the medical image reconstruction task and the medical image segmentation task, which is jointly optimized to improve the segmentation performance. Our MFEL framework is shown in Figure 1, which includes a feature interaction-driven image reconstruction (FIIR) and a feature-embedded representation image segmentation (FRIS). The specific details are as follows.

### 3.1 Feature interaction-driven image reconstruction

FIIR employs self-supervised learning to obtain fine-grained feature representations, thereby enhancing the segmentation feature representations. It consists of three components: foreground image reconstruction (FIR), background image reconstruction (BIR), and medical image reconstruction (MIR). Specifically, FIR generates foreground feature, BIR provides background feature, and MIR obtains fine pixel-level feature. Foreground feature contains the key object information (e.g., edges, textures, structures), and background feature includes the irrelevant environment information. In this way, the two features can enhance the pixel-level fine-grained feature representation during medical image reconstruction.

#### 3.1.1 Foreground and background feature extraction

The medical image *I*_*s*_ first can be divided into foreground image *I*_*f*_ and background image *I*_*b*_ by the segmentation mask *I*_*m*_. *I*_*m*_ labels the foreground information as 1 and the background information as 0. Therefore, *I*_*f*_ and *I*_*b*_ can be formulated as follows:


(1) $$\begin{array}{l} I_{f}=I_{s}⊗I_{m},I_{b}=I_{s}⊗\left(1-I_{m}\right). \end{array}$$

Then, *I*_*f*_ and *I*_*b*_ are respectively fed into the foreground encoder $E_{f}^{i}$ and the background encoder $E_{b}^{i}$ to extract the foreground feature $F_{f}^{i}$ and the background feature $F_{b}^{i}$, where *i* = 1, 2, 3 denotes the layer index. Finally, $F_{f}^{i}$ and $F_{b}^{i}$ are input to the foreground decoder *D*_*f*_ and the background decoder *D*_*b*_ to reconstruct the foreground image $I_{f}^{\prime }$ and the background image $I_{b}^{\prime }$. The self-supervised foreground reconstruction loss *L*_*f*_ and self-supervised background reconstruction loss *L*_*b*_ focus on extracting foreground and background information of the medical segmentation image, which can be formulated as:


(2) $$\begin{array}{l} L_{f}=||I_{f}^{\prime }-I_{f}|{|}_{1},L_{b}=||I_{b}^{\prime }-I_{b}|{|}_{1}, \end{array}$$

where ||·||_1_ represents the *l*_1_ norm.

#### 3.1.2 Pixel-level fine-grained feature generation

**Figure 2:**
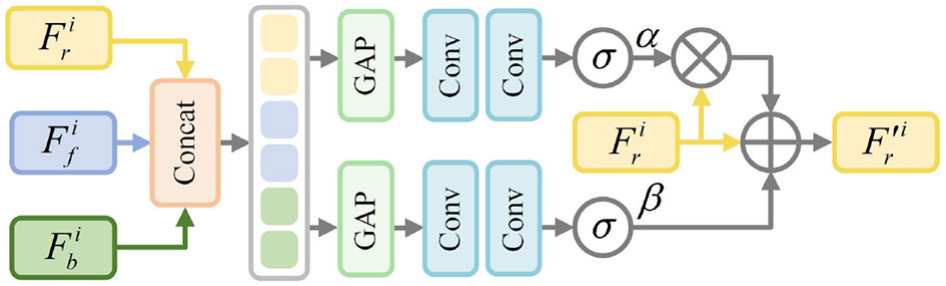
Architecture of adaptive feature modulation module.

As shown in Figure 2, $F_{f}^{i}$ and $F_{b}^{i}$ from each layer are fed into the adaptive feature modulation module $\Phi_{a}^{i}$, thereby helping SIR to obtain more significant foreground and background features. Specifically, $F_{f}^{i}$, $F_{b}^{i}$ and the initial pixel-level feature $F_{r}^{i}$ first perform channel feature concatenation to generate the fusion feature *F*_*u*_, and then the global semantic features are extracted by using global average pooling. Further, we utilize dual-stream convolutional blocks to generate calibration parameters α and β to guide $F_{r}^{i}$. This calibration process can be represented as:


(3) $$\begin{array}{l} {\hat{F}}_{r}^{i}=\left(1+\alpha \right)\times F_{r}^{i}+\beta . \end{array}$$

Next, the calibrated pixel-level fine-grained feature ${\hat{F}}_{r}^{i}$ is fed into the reconstruction decoder to reconstruct the medical image. Finally, the medical image reconstruction loss $L_{s}$ ensures that the pixel-level fine-grained features can reconstruct a complete segmentation image, which can be expressed as follows:


(4) $$\begin{array}{l} L_{s}=||I_{r}^{\prime }-I_{s}|{|}_{1}, \end{array}$$

where *I*_*s*_ denotes a medical image, and $I_{r}^{\prime }$ represents a reconstructed medical image.

### 3.2 Feature representation reinforcement learning

#### 3.2.1 Bi-directional fusion module via hierarchical guidance

**Figure 3:**
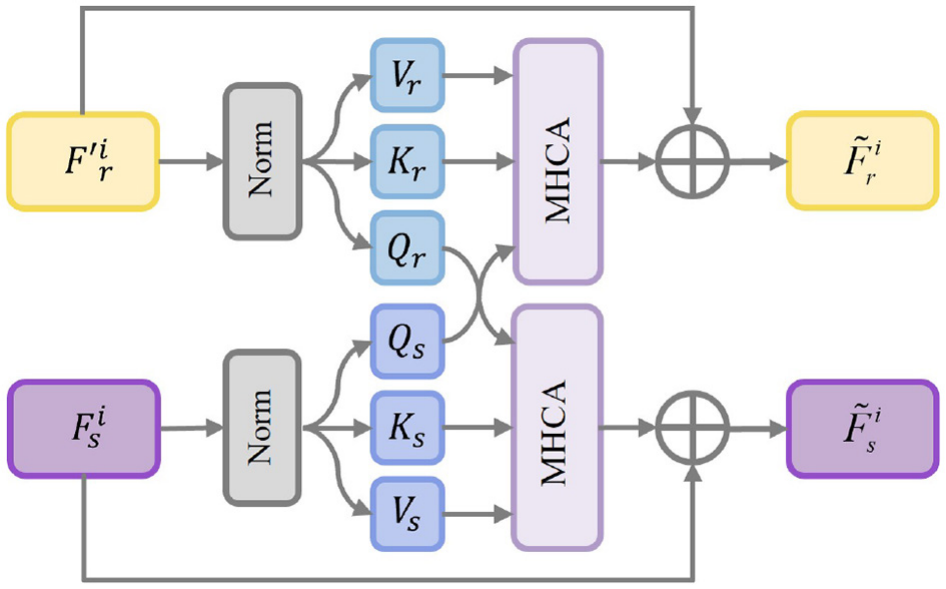
Architecture of bi-directional fusion module.

In Section 3.1, we obtain pixel-level fine-grained feature ${\hat{F}}_{r}^{i}$ from FIIR. Specifically, as shown in Figure 3, *I*_*s*_ is first fed into the segmentation encoder $E_{s}^{i}$ to extract the segmentation semantic feature $F_{s}^{i}$. Then, $F_{s}^{i}$ and ${\hat{F}}_{r}^{i}$ are input to the bi-directional fusion module $\Phi_{b}^{i}$ to obtain a complete feature representation with strong semantics and rich details. Specifically, we compute respectively the cross-attention weights between $F_{s}^{i}$ and ${\hat{F}}_{r}^{i}$, thereby jointly modeling the complementary relationship between the reconstruction branch and the semantic branch. In this process, ${\hat{F}}_{r}^{i}$ uses semantic clues to guide $F_{s}^{i}$ to enhance structural perception, while $F_{s}^{i}$ employs textural details to enhance the spatial resolution of ${\hat{F}}_{r}^{i}$. In particular, First, $F_{s}^{i}$ generates the query vector *Q*_*s*_ and ${\hat{F}}_{r}^{i}$ generates the key-value pair (*K*_*r*_, *V*_*r*_). Similarly, *Q*_*r*_ is obtained via ${\hat{F}}_{r}^{i}$, and (*K*_*s*_, *V*_*s*_) is generated through $F_{s}^{i}$. The two-stream cross-modal attention is computed as follows:


(5) $$\begin{array}{l} {\mathrm{Attn}}_{r}=\mathrm{Softmax}\left(\frac{Q_{s}K_{r}^{⊤}}{\sqrt{d}}\right),{\mathrm{Attn}}_{s}=\mathrm{Softmax}\left(\frac{Q_{r}K_{s}^{⊤}}{\sqrt{d}}\right), \end{array}$$

where $\sqrt{d}$ is the channel dimension of each attention head and *Softmax* is an activation function. Attn_*r*_ denotes the segmentation-guided attention map, and Attn_*s*_ represents the reconstruction-guided attention map. Subsequently, we adopt feature aggregation and residual concatenation to generate two enhanced features ${\tilde{F}}_{r}^{i}$ and ${\tilde{F}}_{s}^{i}$, which can be formulated as:


(6) $$\begin{array}{l} {\tilde{F}}_{r}^{i}=C_{1}^{1}\left({\mathrm{Attn}}_{t}\times V_{r}\right)+{\hat{F}}_{r}^{i},{\tilde{F}}_{s}^{i}=C_{1}^{1}\left({\mathrm{Attn}}_{r}\times V_{s}\right)+{\hat{F}}_{s}^{i}, \end{array}$$

where $C_{1}^{1}$ denotes one convolutional layer with 1 × 1 kernel. Finally, we fuse ${\tilde{F}}_{r}^{i}$ and ${\tilde{F}}_{s}^{i}$ to generate the refined segmentation feature ${\bar{F}}_{s}^{i}$ through concatenation and convolution operations, thus enhancing the feature representation ability.

#### 3.2.2 High-level semantic feature mining via multi-branch vision mamba

**Figure 4:**
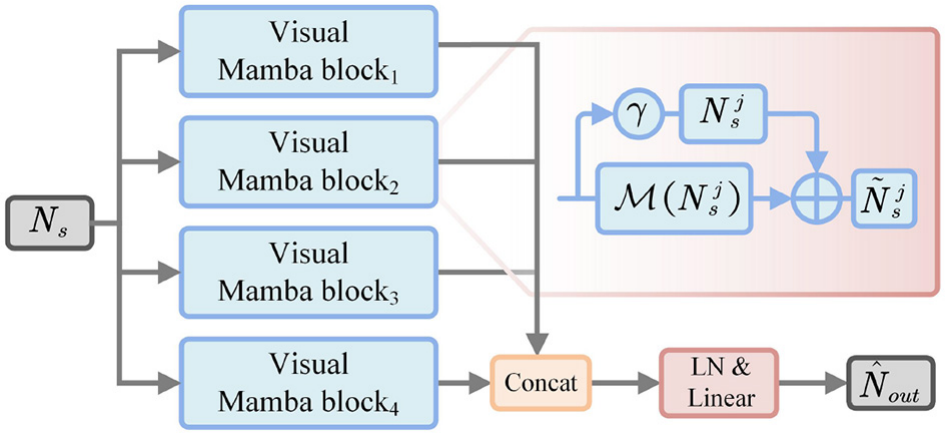
Architecture of multi-branch vision mamba.

Multi-branch vision mamba is constructed to force the model to mine high-level semantic information, thus improving the feature representation. Specifically, as shown in Figure 4, ${\bar{F}}_{s}^{3}$ firstly is fed into *E*_*M*_ to perform flattening and normalization, thereby generating segmentation sequence feature *N*_*s*_. Then, we divide *N*_*s*_ into four groups to learn the important representations of different sub-regions, which can be represented as:


(7) $$\begin{array}{l} \left[N_{s}^{1},N_{s}^{2},N_{s}^{3},N_{s}^{4}\right]=Split\left(N_{s}\right). \end{array}$$

Next, each subsequence is respectively fed into the weight-sharing Mamba module to perform state modeling, and then refine the representation by residual operations:


(8) $$\begin{array}{l} {\tilde{N}}_{s}^{j}=M\left(N_{s}^{j}\right)+\gamma \cdot N_{s}^{j},\;j\in \left\{1,2,3,4\right\} \end{array}$$

where γ is a scaling factor. M(·) denotes the Mamba function. Then, the updated subsequence *N*_*j*_ is performed to feature concatenation from the channel dimension, thus generating the enhanced sequence representation:


(9) $$\begin{array}{l} {\tilde{N}}_{s}=Concat\left({\tilde{N}}_{s}^{1},{\tilde{N}}_{s}^{2},{\tilde{N}}_{s}^{3},{\tilde{N}}_{s}^{4}\right). \end{array}$$

where *Concat*(·) denotes the feature concatenation operation. Subsequently, ${\tilde{N}}_{s}$ is normalized and linearly transformed to project to the original feature dimension, which can be expressed as:


(10) $$\begin{array}{l} {\hat{N}}_{out}=Proj\left(LN\left({\tilde{N}}_{s}\right)\right), \end{array}$$

where *LN*(·) represents layer normalization, and *Proj*(·) indicates linear projection.

Therefore, we utilized the state-space mechanism of Mamba to capture long-distance contextual information. Then, multi-branch decomposition is used to enhance the feature representation between different sub-regions. In this way, multi-branch vision mamba establishes the dependency between global semantics and local details, thus helping the model to improve the segmentation accuracy of key objects.

### 3.3 Model training

#### 3.3.1 Image reconstruction head

To constrain the difference at the pixel level between the reconstructed image and the segmentation image, the image reconstruction head *D*_*f*_, *D*_*r*_ and *D*_*b*_ adopt the reconstruction loss $L_{rec}$, which can be defined as follows:


(11) $$\begin{array}{l} L_{rec}=L_{s}+L_{f}+L_{b}, \end{array}$$

where $L_{s}$, $L_{f}$ and $L_{b}$ denote foreground reconstruction loss, background reconstruction loss and medical image reconstruction loss, respectively.

#### 3.3.2 Semantic segmentation head

The BCE loss $L_{bce}$ aims to predict the per-pixel classification accuracy. The Dice loss $L_{dice}$ can measure the overall overlap region between the prediction mask $I_{s}^{\prime }$ and the ground truth *I*_*gt*_. Thus, we jointly $L_{bce}$ and $L_{dice}$ constrain the segmentation head *S*_*h*_, which can be expressed as:


(12) $$\begin{array}{l} L_{mask}=L_{bce}\left(I_{s}^{\prime },I_{gt}\right)+L_{dice}\left(I_{s}^{\prime },I_{gt}\right). \end{array}$$

Finally, the total training loss can be expressed as:


(13) $$\begin{array}{l} L_{total}=L_{rec}+L_{mask}. \end{array}$$

## 4 Experiments

In this section, we present a comprehensive overview of our experiments. We begin by introducing the datasets used in the study, followed by detailed descriptions of the experimental settings and implementation details. We then report the results of comparison experiments against state-of-the-art methods. In addition, we perform ablation studies to assess the impact of each key component. These experiments are designed to validate the effectiveness of the proposed method and to provide insights into the contribution of different modules to the overall performance.

### 4.1 Experimental settings

#### 4.1.1 Datasets

**GLAS** (57) dataset consists of 165 microscopy images of colorectal adenocarcinoma tissue sections at stage T3 or T4, stained with H&E. Each image has a resolution of 128 × 128 pixels and is collected from a different patient. Due to variations in cancer progression, the lesions exhibit significant differences in shape and distribution. Meanwhile, since all samples originate from the same type of tissue, the surrounding environments are relatively consistent. Additionally, some cells are damaged or ruptured during the sampling process, resulting in large inter-cell variability. These factors make the dataset highly challenging. According to the official split, the training set contains 85 images and the test set contains 80 images. This dataset is mainly used to assess the model's capability in segmenting dense lesion regions and small targets.

**ISIC2016** **(****58****)** and **ISIC2017** **(****59****)** datasets were released by the International Skin Imaging Collaboration (ISIC) in 2016 and 2017, respectively. They were used as official datasets for the skin lesion analysis challenges held in those years. The goal of these datasets is to raise global awareness of skin disease diagnosis and to improve the detection of melanoma and other benign or malignant lesions. Both datasets contain a large number of samples and include various types of skin lesions. Due to the diversity of lesion types and the wide range of patient backgrounds, the samples show high variability in texture, color, and structure. In addition, some mild lesions look very similar to normal skin, which makes it hard to identify lesion boundaries. This increases the difficulty of the segmentation task. In this study, we evaluate the segmentation performance of our model using the ISIC2016 and ISIC2017 datasets. Both datasets follow the official training and testing splits: ISIC2016 includes 900 training images and 379 testing images, while ISIC2017 consists of 2,000 training images and 600 testing images. All images are resized to 256 × 256 pixels to ensure consistency during the experiments.

**PH2** **(****60****)** is a public dataset designed for dermoscopic image segmentation and classification. It aims to support research on computer-aided diagnosis of melanocytic lesions. The images were collected at the dermatology department of Pedro Hispano Hospital in Portugal. All images were captured under the same conditions using the Tuebinger mole analyzer system with 20 × magnification. The dataset contains 200 dermoscopic images of melanocytic lesions, including 80 common nevi, 80 atypical nevi, and 40 melanomas. PH2 serves as a reliable benchmark for evaluating lesion detection, segmentation, and classification algorithms. In our experiments, all images were resized to 256 × 256 pixels. It is worth noting that we used PH2 as an external validation dataset. We tested it directly using the model trained on ISIC2016 to assess the effectiveness of our method and its potential for future clinical applications.

#### 4.1.2 Metrics

In the quantitative analysis, we adopt widely used evaluation metrics in the field of medical image segmentation. Specifically, we use Precision, Recall, F1, and Intersection over Union (IoU) to assess the performance of the proposed model. Here, TP denotes true positives, FP denotes false positives, TN denotes true negatives, and FN denotes false negatives. These metrics jointly provide a comprehensive evaluation of the model's accuracy and completeness from multiple perspectives.

Precision measures the proportion of true positives among all regions predicted as positive (e.g., lesion areas). It reflects the model's ability to control FP. In medical image segmentation, a high Precision means the model can avoid wrongly identifying normal areas as lesions, which helps reduce the risk of misdiagnosis. When Precision is high, most of the predicted lesion regions are actually correct, and the FP rate is low. This is especially important in cases with small lesions or strong background noise. In such situations, Precision is a key metric to evaluate how well the model limits over-segmentation. The formula is given as:


(14) $$\begin{array}{l} \text{Precision}=\frac{TP}{TP+FP} \end{array}$$

Recall measures the model's ability to detect all positive targets. It shows how many of the actual positive pixels are correctly identified. In medical image segmentation, a high Recall means the model can successfully detect most lesion areas, which helps reduce missed detections and is important for clinical diagnosis support. The formula is given as:


(15) $$\begin{array}{l} \text{Recall}=\frac{TP}{TP+FN} \end{array}$$

F1 is the harmonic mean of Precision and Recall. It is used to evaluate both the accuracy and completeness of the model. When there is a large gap between Precision and Recall, F1 provides a more balanced result. In segmentation tasks, F1 is especially useful for assessing model performance under class imbalance, such as small lesions against large background regions. A higher F1 indicates that the model achieves a good balance between accuracy and completeness. The formula is given as:


(16) $$\begin{array}{l} \text{F1}=\frac{2\cdot \text{Precision}\cdot \text{Recall}}{\text{Precision}+\text{Recall}}=\frac{2TP}{2TP+FP+FN} \end{array}$$

IoU is one of the most widely used metrics in image segmentation. It measures the overlap between the predicted region and the GT. It is defined as the ratio of the intersection area to the union area of the prediction and the GT. IoU directly reflects how well the predicted boundary matches the actual boundary. A higher IoU means the predicted region aligns more closely with the GT, indicating better segmentation accuracy. Compared to F1, IoU is more sensitive to small differences and is suitable for evaluating boundary localization. The formula is given as:


(17) $$\begin{array}{l} \text{IoU}=\frac{TP}{TP+FP+FN} \end{array}$$

#### 4.1.3 Implementations

We use NVIDIA GeForce RTX 4090 GPU to train and inference the model. The network framework is Pytorch. EPOCH is set to 150, and Batch is 4. The optimizer is Adam that uses momentum strategy to steadily update the model parameters. We employ warm-up and cosine annealing schedulers to achieve slow startup in the early stages and fine convergence in the later. The initial learning rate is 1e-3 and gradually decays to 1e-5.

### 4.2 Comparison with SOTA methods

To ensure a more comprehensive and reliable evaluation of model performance, we compare our method against state-of-the-art (SOTA) approaches from the past four years across different network architectures. These comparisons highlight the advantages of our model. Specifically, we select representative CNN-based methods including MsRED (25), MFSNet (11), DCSAU (27), and U-Net V2 (28); Transformer-based methods including BAT (30), FAT-Net (31), SSFormer (32), and CASF-Net (36); and a recent Mamba-based method, U-vm-unet (52). Extensive comparisons are conducted on four public datasets.

#### 4.2.1 GLAS

##### 4.2.1.1 Qualitative comparisons

As in Table 1, we compare several representative methods from recent years on the GLAS dataset. The results show that Ours achieves the highest scores in F1, IoU, and Precision, and ranks second in Recall, slightly behind MFSNet. Ours reaches 82.07 in IoU, which shows a clear advantage over other methods. This indicates that the predicted lesion regions by Ours have better overlap with the GT and more accurate boundary localization. The Precision score reaches 91.01, suggesting that Ours effectively reduces false positives, which is important in scenarios where over-segmentation should be avoided. Considering that the GLAS dataset contains complex gland structures, a high proportion of small targets, and blurry boundaries, IoU and Precision are key metrics to evaluate real segmentation quality. Some methods achieve higher Recall but perform worse in IoU and Precision, which may be caused by over-segmentation. In contrast, Ours maintains high Recall while achieving high accuracy, showing strong boundary modeling ability and overall robustness.

**Table 1:** Qualitative comparison results on the GLAS dataset.

**Method**	**F1(%)**	**IoU(%)**	**Precision(%)**	**Recall(%)**
BAT (30)	83.93	73.47	84.85	84.43
FAT-Net (31)	86.45	76.14	88.23	84.75
MsRED (25)	85.92	75.32	87.20	84.69
MFSNet (11)	86.33	75.95	81.70	89.20
SSFormer (32)	71.60	59.13	74.17	74.00
CASF-Net (36)	85.83	76.08	88.05	75.20
DCSAU (27)	88.28	79.03	87.67	88.32
U-vm-unet (52)	82.07	69.60	74.39	86.60
U-Net V2 (28)	88.90	80.86	87.31	89.16
Ours	89.73	82.07	91.01	89.18

Red indicates the best performance, and blue indicates the second best.

##### 4.2.1.2 Quantitative comparisons

**Figure 5:**
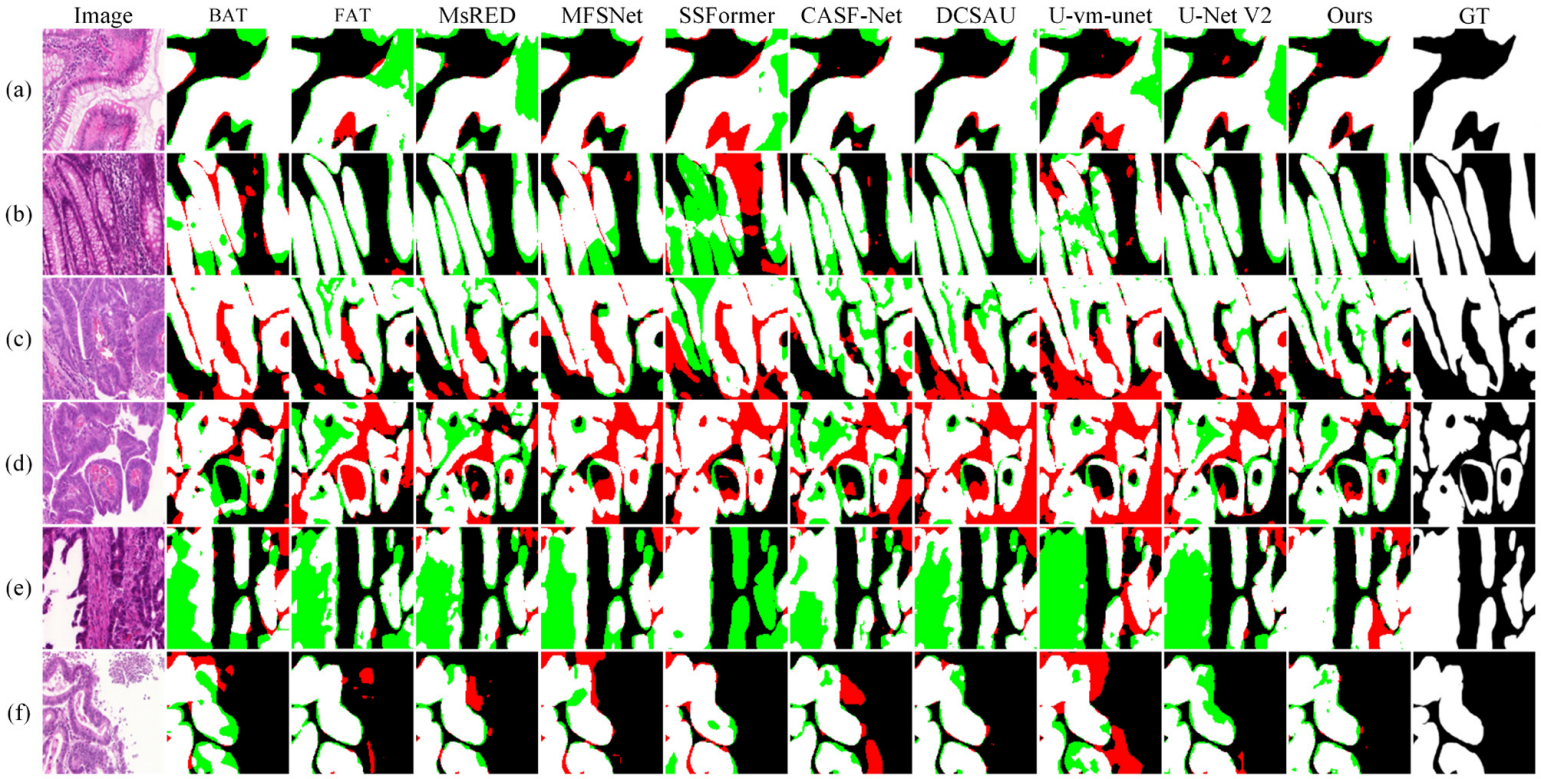
Quantitative comparison results on the GLAS dataset. Green regions indicate areas missed with respect to the GT, while red regions represent incorrectly predicted areas compared to the GT.

Figure 5 shows the visual comparison results on the GLAS dataset. In sample (a), the lesion cell has clear boundaries and appears hollow due to structural damage. SSFormer and U-vm-unet make obvious errors in this case, leading to inaccurate boundary prediction and incorrect segmentation of the cell structure. In samples (c) and (d), the lesion boundaries are blurry. Most methods fail to extract the target contours correctly and show severe missegmentation. In contrast, although Ours also shows some boundary errors, it preserves the overall shape of the target more completely.

In samples (e) and (f), the white regions represent the internal cell structure and the external background, respectively. These two samples come from different experimental conditions. For sample (e), methods like U-Net V2 miss part of the structure on the left side and mistakenly classify it as background. In sample (f), these methods show incomplete cell boundaries. In comparison, Ours gives results that are closer to the ground truth in both samples, showing better generalization. However, it is worth noting that Ours still makes a mistake in identifying the cell on the right side of sample (e), which suggests that there is still room to improve robustness across different environments.

**Figure 6:**
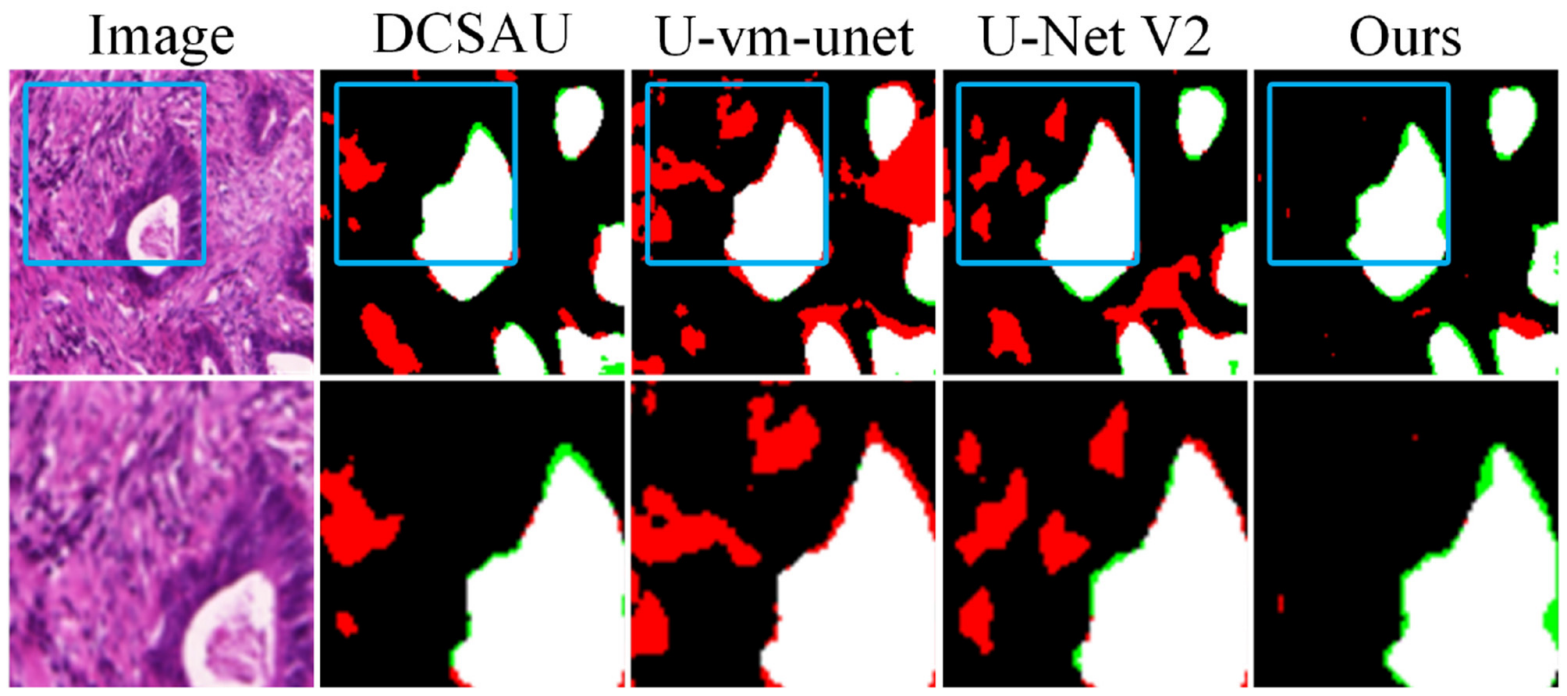
Detail comparison on the GLAS dataset.

To evaluate model performance on small targets and in noisy environments, we conducted local zoom-in comparisons on representative samples, as shown in Figure 6. DCSAU, U-vm-unet, and U-Net V2 often misidentify background textures as lesion regions, especially when boundaries are blurred or targets are irregular. This suggests limited robustness to noise and weak discrimination in challenging cases. In contrast, Ours better distinguishes true lesions from noisy backgrounds and successfully detects small, low-contrast targets. Despite minor boundary errors, it shows stronger resistance to noise and improved sensitivity to fine-grained lesion structures.

#### 4.2.2 ISIC2016

##### 4.2.2.1 Qualitative comparisons

On the ISIC2016 dataset, we compare our method with several representative approaches from recent years and evaluate segmentation performance from multiple aspects. As in Table 2, Ours achieves the best results across all four metrics: F1, IoU, Precision, and Recall, demonstrating strong overall performance. Specifically, the F1 reaches 94.14 and the IoU reaches 89.40, which shows a clear improvement over other methods. This indicates that our model provides a better balance between segmentation accuracy and region coverage, and can more precisely recover lesion shapes. The Precision reaches 95.48, showing stable control over false positives and helping reduce the misclassification of normal skin areas. The Recall reaches 93.45, ensuring high detection rates for lesion regions, which is important in clinical settings where missed detections must be minimized. The ISIC2016 dataset contains many benign and malignant skin lesions with blurry boundaries and similar textures, making segmentation more challenging. Compared to Ours, U-Net V2 achieves a similar Recall but lower Precision, which may cause over-segmentation. DCSAU shows good Precision, but its Recall is lower, which leads to missed lesion areas. In contrast, Ours maintains a better balance across all four metrics, indicating stronger segmentation ability under challenges such as background similarity, boundary ambiguity, and class imbalance commonly found in dermoscopic images.

**Table 2:** Qualitative comparison results on the ISIC2016 dataset.

**Method**	**F1(%)**	**IoU(%)**	**Precision(%)**	**Recall(%)**
BAT (30)	91.22	84.99	93.36	91.32
FAT-Net (31)	91.58	85.42	92.36	92.79
MsRED (25)	91.61	85.51	93.37	91.90
MFSNet (11)	92.57	86.17	93.85	91.33
SSFormer (32)	91.37	85.63	90.18	93.22
CASF-Net (36)	91.46	85.50	92.26	88.22
DCSAU (27)	92.72	86.42	91.42	94.05
U-vm-unet (52)	92.79	86.54	93.92	91.68
U-Net V2 (28)	93.02	86.96	96.83	93.14
Ours	94.14	89.40	95.48	93.45

Red indicates the best performance, and blue indicates the second best.

##### 4.2.2.2 Quantitative comparisons

**Figure 7:**
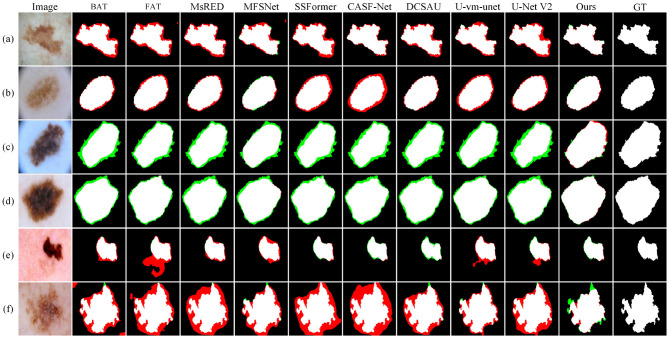
Quantitative comparison results on the ISIC2016 dataset. Green regions indicate areas missed with respect to the GT, while red regions represent incorrectly predicted areas compared to the GT.

Figure 7 presents the visual comparison results on the ISIC2016 dataset. In clinical diagnosis, accurate boundary identification of lesions is important for evaluating the development stage and malignancy of the disease. However, in samples (a)–(d), many baseline methods show varying degrees of boundary errors, such as incomplete contours or blurred edges. In contrast, Ours performs more consistently in boundary modeling and produces results that are closer to the ground truth, which is of higher clinical value.

In sample (e), there is a dark skin area in the lower left region with texture similar to the lesion. FAT, U-vm-unet, and U-Net V2 all misclassify this area as a lesion, resulting in obvious false segmentation. MFSNet successfully captures the main region but misses parts near the boundary, which affects the overall contour quality.

Sample (f) contains a lesion with complex boundaries and fine internal structure. The lesion is located near the image edge, and the background interference is strong. These factors make boundary detection more difficult. Most baseline methods show shifted or broken contours in this case. Although Ours also makes some errors, its prediction is still the closest to the ground truth and better preserves both the overall shape and boundary continuity.

**Figure 8:**
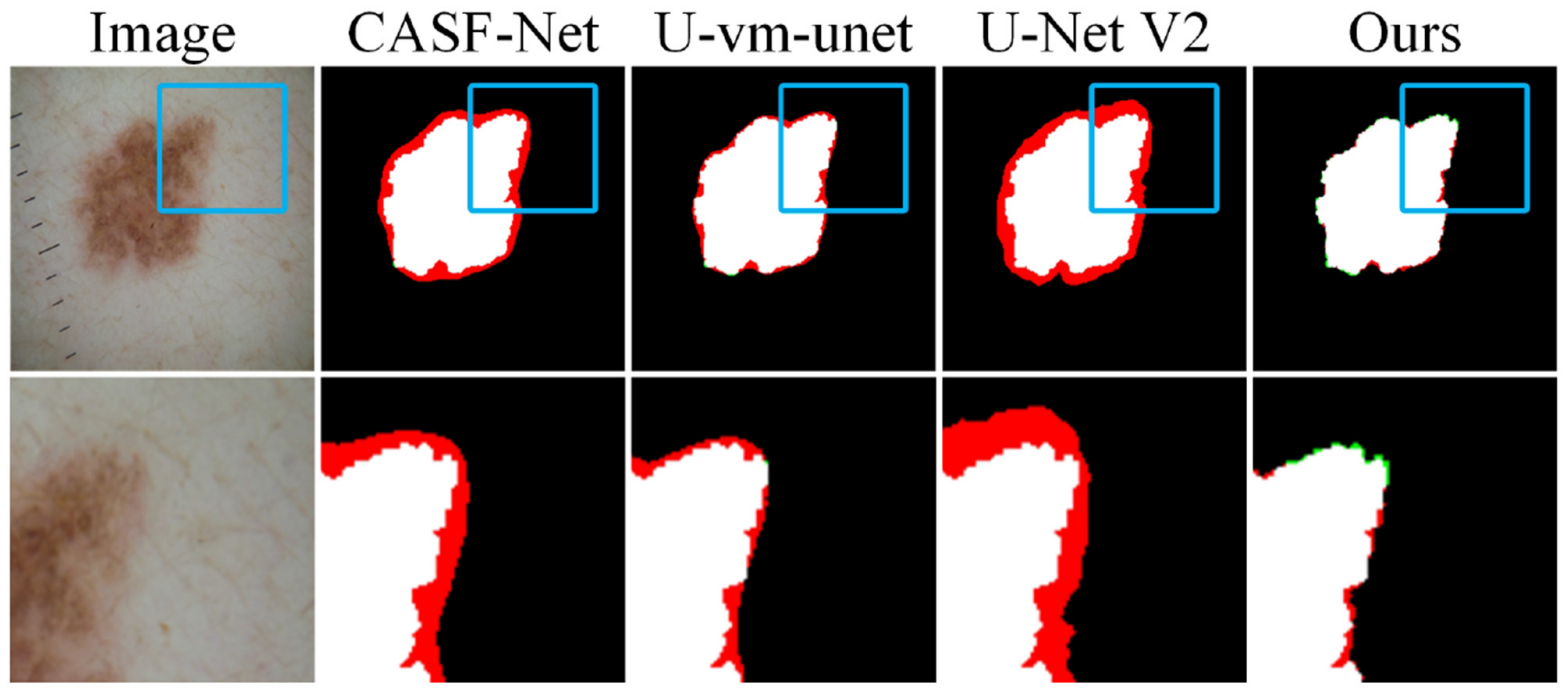
Detail comparison on the ISIC2016 dataset.

To further evaluate the model's ability to handle blurry boundaries, we selected a group of representative samples and performed local zoom-in comparisons, as shown in Figure 8. The results show that CASF-Net and U-Net V2 produce relatively coarse boundary predictions. Their outputs often show broken or expanded contours, which do not match the ground truth accurately. In contrast, Ours shows better alignment with the ground truth boundaries and performs more stably in preserving fine structural details. These results further demonstrate that our method has stronger fine-grained boundary perception and achieves higher localization accuracy for targets with unclear edges.

#### 4.2.3 PH2

##### 4.2.3.1 Qualitative comparisons

Table 3 shows the test results on the PH2 dataset, which is used as an external validation set. The model is trained on the ISIC2016 dataset. As shown, Ours achieves the highest scores in the two key metrics, F1 and IoU, with values of 94.44 and 89.70 respectively. These results clearly outperform other methods and demonstrate strong overall segmentation ability and good generalization performance across datasets.

**Table 3:** Qualitative comparison results on the PH2 dataset.

**Method**	**F1(%)**	**IoU(%)**	**Precision(%)**	**Recall(%)**
BAT (30)	89.24	81.62	96.33	84.99
FAT-Net (31)	90.66	83.54	86.13	97.14
MsRED (25)	88.61	80.65	84.35	95.97
MFSNet (11)	91.42	84.19	89.12	93.84
SSFormer (32)	90.77	83.98	89.04	94.65
CASF-Net (36)	90.85	83.86	86.92	96.60
DCSAU (27)	87.33	77.51	90.27	84.58
U-vm-unet (52)	86.87	76.79	86.43	87.32
U-Net V2 (28)	90.70	82.98	92.88	95.28
Ours	94.44	89.70	93.73	95.37

Red indicates the best performance, and blue indicates the second best.

Although the Recall of Ours is not the highest among all methods, it remains at a high level. It is worth noting that the Recall of Ours is slightly lower than that of FAT-Net's 97.14 and CASF-Net's 96.60. This may be due to the fact that lesions in the PH2 dataset are more regular in shape and have relatively clearer boundaries. FAT-Net and CASF-Net tend to enlarge the predicted regions to increase the recall rate. However, this strategy often leads to lower precision and causes a drop in both IoU and F1. In contrast, Ours keeps a good balance. It maintains a reasonable recall while avoiding over-segmentation, which helps improve boundary accuracy and overall model stability.

##### 4.2.3.2 Quantitative comparisons

**Figure 9:**
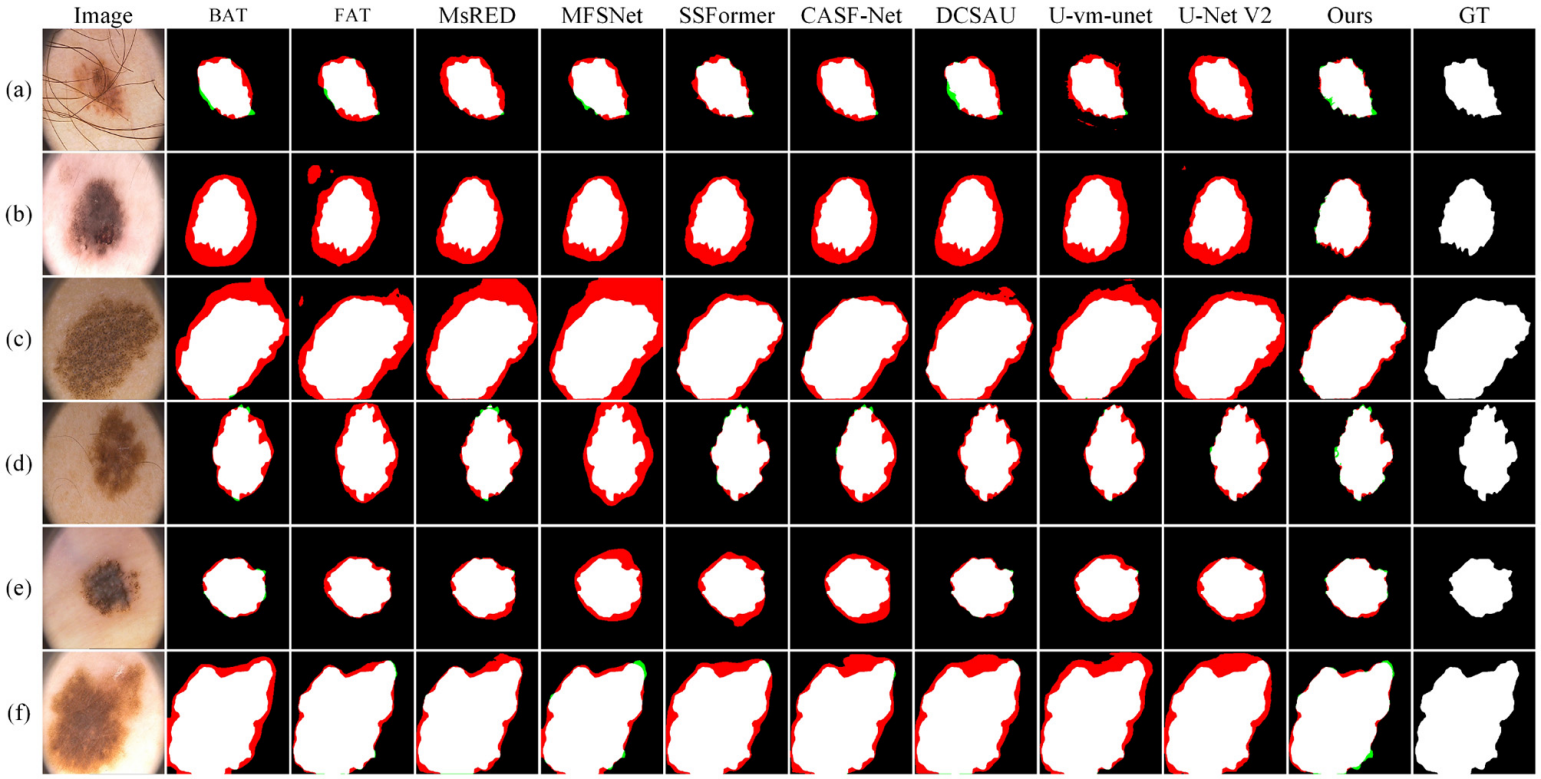
Quantitative comparison results on the PH2 dataset. Green regions indicate areas missed with respect to the GT, while red regions represent incorrectly predicted areas compared to the GT.

Figure 9 shows the segmentation results of several samples from the PH2 dataset. Overall, most methods can outline the general shape of the lesion, but there are still clear differences in boundary details and the handling of interference regions. In samples (a) and (b), where the lesion boundaries are relatively clear, U-Net V2 and CASF-Net produce coarser edges. In contrast, Ours generates contours that better align with the ground truth, with smoother and more complete boundaries, especially in the transition areas around the lesion. In sample (f), the lesion is large and structurally complex. Methods such as BAT and U-Net V2 show varying degrees of over-segmentation, with a large number of false positive areas (in red). Although Ours also has some prediction errors, its boundaries are more compact and the over-segmentation is significantly reduced.

**Figure 10:**
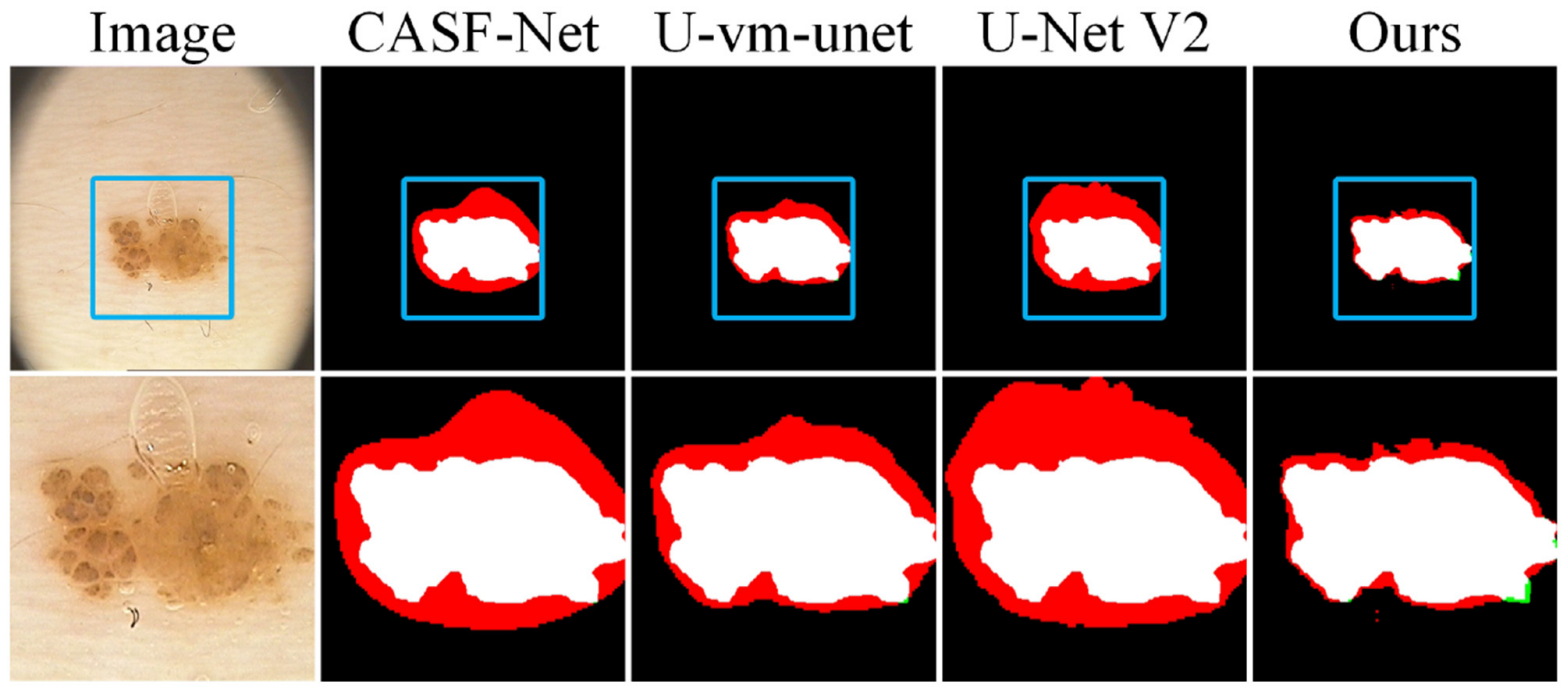
Detail comparison on the PH2 dataset.

We also select a group of samples for local zoom-in comparison, as shown in Figure 10. In these samples, the lesion regions are located within a liquid environment, and bubbles above the lesions introduce interference. This causes CASF-Net, U-vm-unet, and U-Net V2 to produce severe misclassifications. Although Ours also shows some boundary inaccuracies due to the blurred edges, its prediction remains the closest to the ground truth.

#### 4.2.4 ISIC2017

##### 4.2.4.1 Qualitative comparisons

Table 4 shows the evaluation results on the ISIC2017 dataset. Ours ranks first in three key metrics: F1, IoU, and Recall, with scores of 88.10, 80.06, and 94.84, respectively. These results show that our model achieves strong overall segmentation quality and high lesion detection sensitivity. In particular, the Recall score is significantly higher than other methods, indicating that our model is more sensitive to lesion regions and can reduce missed detections. This is useful for clinical applications that require high recall. Compared to methods such as U-Net V2 and MFSNet, Ours maintains a high Recall while achieving a better balance in IoU and F1, showing better boundary modeling ability and practical value.

**Table 4:** Qualitative comparison results on the ISIC2017 dataset.

**Method**	**F1(%)**	**IoU(%)**	**Precision(%)**	**Recall(%)**
BAT (30)	84.85	76.23	86.64	88.75
FAT-Net (31)	84.79	76.06	89.08	85.93
MsRED (25)	84.43	75.79	91.21	83.61
MFSNet (11)	85.42	74.55	91.91	79.79
SSFormer (32)	83.43	71.30	81.51	85.54
CASF-Net (36)	84.20	72.71	85.14	84.51
DCSAU (27)	85.92	75.32	83.93	88.01
U-vm-unet (52)	85.26	74.93	89.51	81.39
U-Net V2 (28)	85.00	73.90	96.26	82.86
Ours	88.10	80.06	83.13	94.84

Red indicates the best performance, and blue indicates the second best.

However, in terms of Precision, Ours performs relatively lower, with a score of 83.13, which is clearly below methods like U-Net V2's 96.26 and MFSNet's 91.91. The ISIC2017 dataset contains more complex lesions with blurry boundaries and irregular shapes. While trying to capture lesion regions more completely, the model may also include neutral areas near the lesion boundary or non-lesion areas with similar appearance. This increases the false positive rate and leads to a lower Precision score.

##### 4.2.4.2 Quantitative comparisons

**Figure 11:**
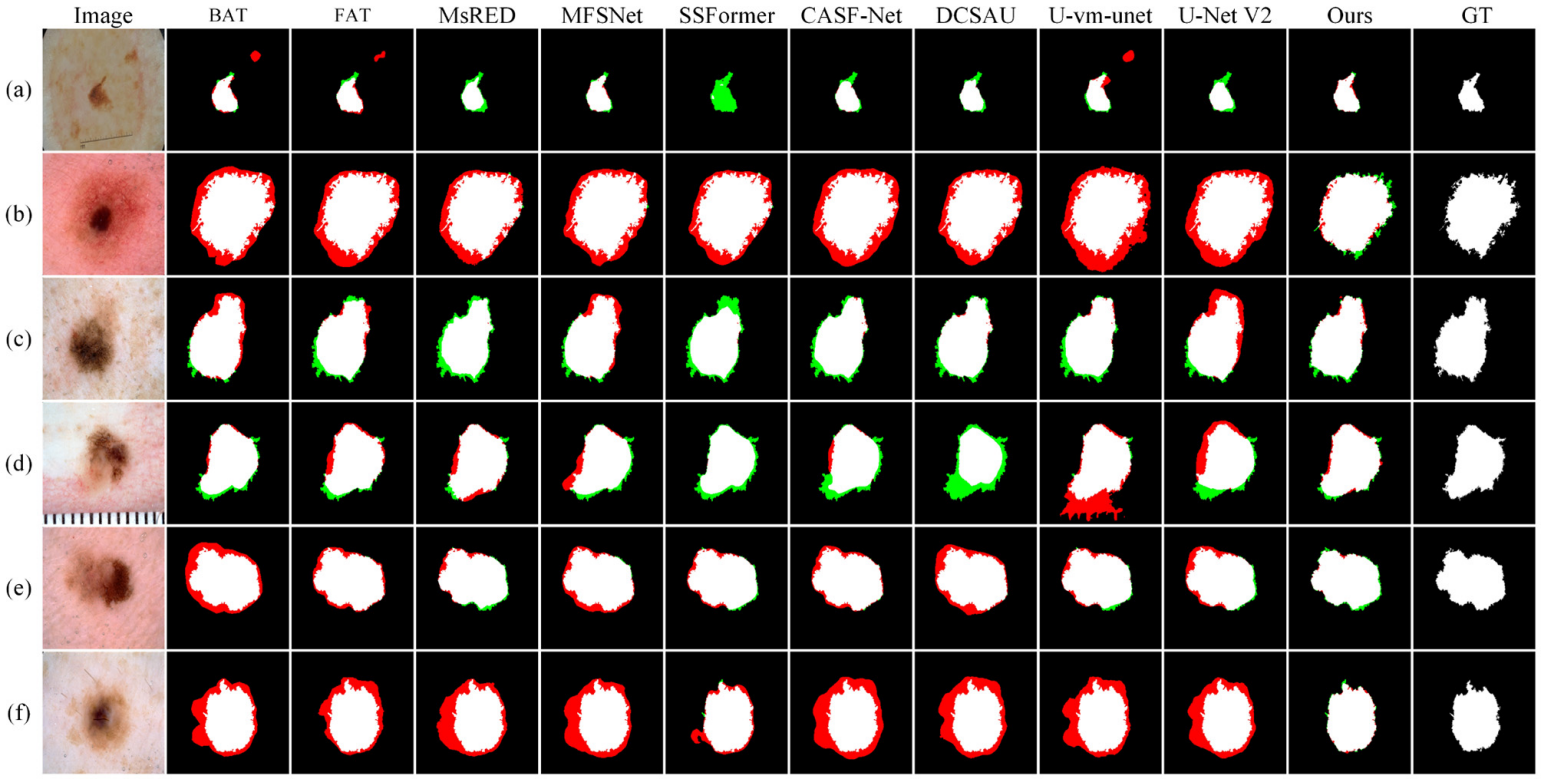
Quantitative comparison results on the ISIC2017 dataset. Green regions indicate areas missed with respect to the GT, while red regions represent incorrectly predicted areas compared to the GT.

As shown in Figure 11, the samples in the ISIC2017 dataset often have more blurred boundary information. This leads to boundary prediction errors across all compared methods. In sample (b), the lesion boundaries are highly similar to the surrounding skin texture, causing all models to misidentify the boundary. In sample (d), although the lesion boundary is relatively clear, the surrounding skin is more complex. As a result, U-vm-unet mistakenly includes the ruler at the bottom as part of the lesion. In sample (e), the lesion gradually darkens from left to right. Most methods can accurately detect the boundary on the right where the contrast is high, but fail to identify the blurry boundary on the left. In contrast, Ours achieves a result that is closest to the ground truth.

**Figure 12:**
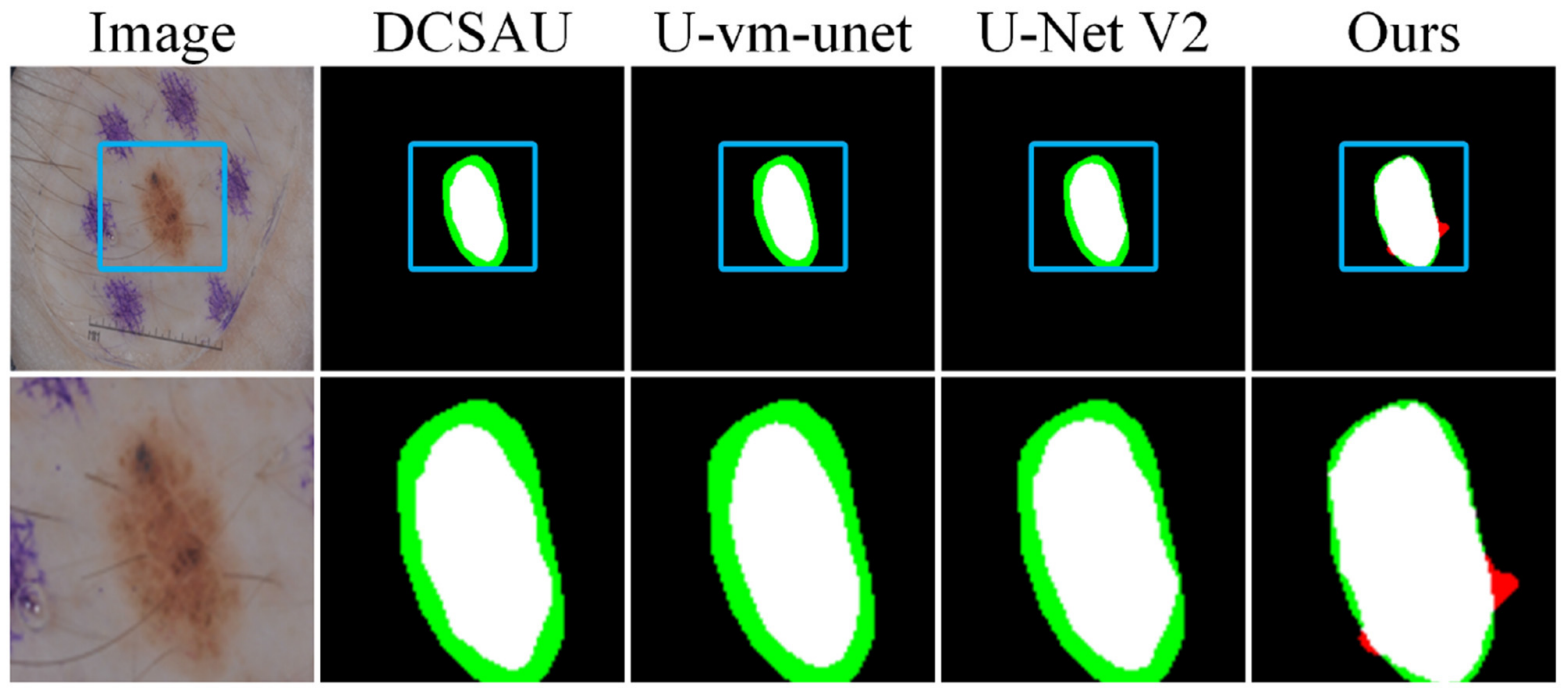
Detail comparison on the ISIC2017 dataset.

In Figure 12, we present a local zoom-in comparison. The blurred and small-sized lesion increases the difficulty of segmentation. Compared with DCSAU and two other methods, Ours shows better performance in boundary prediction. However, Ours is still affected by the surrounding environment and mistakenly identifies hair in the lower-left area as part of the boundary. This indicates that there is still room for improvement in handling fine-grained features.

### 4.3 Ablation studies

#### 4.3.1 GLAS

**Figure 13:**
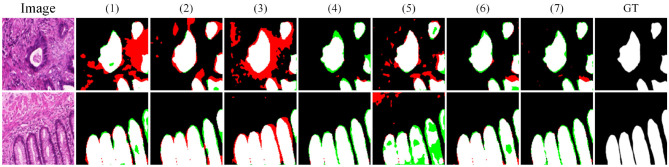
Quantitative comparative results of ablation experiments on the GLAS dataset.

To evaluate the contribution of each module in the model, we conducted a systematic ablation study on the GLAS dataset. The quantitative results obtained after removing different components are presented in Table 5, and the corresponding visual segmentation outputs are shown in Figure 13, offering a clear view of how each module affects the final performance.

**Table 5:** Ablation studies results on the GLAS dataset.

**Method**	**F1(%)**	**IoU(%)**	**Precision(%)**	**Recall(%)**
(1) w/o FIIR	79.68	67.90	81.94	80.65
(2) w/o $\Phi_{a}^{i}$ summary	84.58	75.42	78.18	96.10
(3) w/o $\Phi_{a}^{i}$ concat	76.27	64.35	66.37	96.31
(4) w/o $\Phi_{b}^{i}$ summary	88.64	80.31	89.77	87.79
(5) w/o $\Phi_{b}^{i}$ concat	89.60	81.91	89.87	88.72
(6) w/o *E*_*M*_	85.01	75.05	84.18	87.77
(7) Ours	89.73	82.07	91.01	89.18

Red indicates the best performance, and blue indicates the second best.

As can be seen from the visual results, w/o FIIR (1) leads to evident deficiencies along the object boundaries and causes incomplete structural predictions. This demonstrates that FIIR plays an important role in enhancing pixel-level detail and supporting the extraction of informative segmentation features. When the adaptive feature modulation module $\Phi_{a}^{i}$ is removed, and then replaced with feature summary (2) or concat (3), the model tends to produce over-segmentation. This is reflected in the increased number of false positives in the predicted maps. Although the recall remains relatively high, reaching 96.10 and 96.31 respectively, the precision drops significantly to 78.18 and 66.37, suggesting that the model becomes less capable of regulating foreground and background responses effectively.

Moreover, we adopt summary (4) and concat (5) operations to replace the bi-directional fusion module $\Phi_{b}^{i}$. The predicted structures remain mostly intact, but the boundaries are less precise, indicating that this module still contributes to enhancing local detail and structural consistency. Further, the removal of the multi-branch vision mamba module *E*_*M*_ (6) results in a decrease in both IoU and F1, and the predicted boundaries become less distinct. This shows that *E*_*M*_ plays a critical role in aggregating hierarchical features and is particularly helpful in capturing complex object shapes.

Among all the configurations, the complete model (7) achieves the best overall performance. It obtains an F1 of 89.73, an IoU of 82.07, a precision of 91.01, and a recall of 89.18. Its visual results are also the most aligned with the ground truth annotations. These observations confirm that the synergy between the proposed modules leads to significant improvements in both segmentation accuracy and visual quality.

#### 4.3.2 ISIC2016

**Figure 14:**
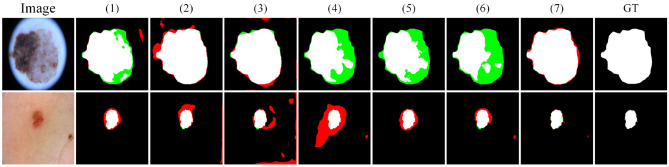
Quantitative comparative results of ablation experiments on the ISIC2016 dataset.

We further validate the effect of each model component by conducting ablation experiments on the ISIC2016 dataset. Table 6 reports the numerical performance under different ablation settings, while Figure 14 illustrates the corresponding segmentation outputs for visual comparison. w/o FIIR (1) leads to a noticeable decline in IoU and F1, which drops to 84.33 and 90.81, respectively. Despite the recall and precision being reasonably balanced, the visual outputs exhibit weaker boundary fidelity, particularly in areas with low contrast, where the predicted masks tend to deviate from the lesion margins. Interestingly, we adopt w/o $\Phi_{a}^{i}$ concat (3) to produce the highest recall at 98.48, suggesting that the model becomes more permissive in capturing lesion pixels. However, this also comes at the cost of increased false positives, as reflected in the relatively lower precision and the presence of redundant red areas in the predicted masks. w/o $\Phi_{a}^{i}$ summary (2) causes the prediction accuracy to decrease, reinforcing that the absence of the modulation structure compromises the foreground-background balancing mechanism.

**Table 6:** Ablation studies results on the ISIC2016 dataset.

**Method**	**F1(%)**	**IoU(%)**	**Precision(%)**	**Recall(%)**
(1) w/o FIIR	90.81	84.33	91.76	91.87
(2) w/o $\Phi_{a}^{i}$ summary	91.64	85.45	86.82	97.09
(3) w/o $\Phi_{a}^{i}$ concat	93.75	88.86	89.19	98.48
(4) w/o $\Phi_{b}^{i}$ summary	91.51	85.14	95.4895.48	88.16
(5) w/o $\Phi_{b}^{i}$ concat	88.77	81.19	89.25	91.17
(6) w/o *E*_*M*_	90.79	84.04	95.23	87.37
(7) Ours	94.14	89.40	95.48	93.45

Red indicates the best performance, and blue indicates the second best.

To verify the effectiveness of the bi-directional fusion module $\Phi_{b}^{i}$, we use $\Phi_{b}^{i}$ summary (4) and $\Phi_{b}^{i}$ concat (4) instead of $\Phi_{b}^{i}$. Specifically, w/o $\Phi_{b}^{i}$ concat (5) has a clearer negative effect, with IoU decreasing to 81.19, accompanied by more pronounced boundary irregularities in the visualization. w/o *E*_*M*_ on IoU and F1 metrics scores lower than the full model. This suggests that although the primary structure still functions, the lack of high-low feature interaction leads to reduced segmentation confidence near ambiguous regions. With all components intact, the full model (7) achieves the strongest performance across all metrics F1 reaches 94.14, IoU improves to 89.40, and both precision and recall are maximized. The output masks are tightly aligned with the lesion contours, even under challenging conditions such as blurry or low-contrast boundaries, confirming the complementary nature of all proposed modules.

## 5 Conclusion

In this paper, we propose a multi-interactive feature embedding learning method for medical image segmentation. The core idea is to establish information interaction between the reconstruction task and the segmentation task, thus achieving superior segmentation performance. Specifically, an adaptive feature modulation module can efficiently fuse foreground and background features, thereby extracting pixel-level fine-grained features. Then, a bi-directional fusion module integrates important feature information between two different tasks, enhancing semantic understanding and detail retention. Finally, a multi-branch visual mamba effectively captures structural details by extracting multi-scale features in parallel, thus improving the model capability in terms of local texture and global semantics. Extensive experiments demonstrate that the proposed method can accurately segment the lesion region compared to other state-of-the-art segmentation methods.

## Data Availability

The original contributions presented in the study are included in the article/supplementary material, further inquiries can be directed to the corresponding author.
